# Supplementary figures and images for: A damped oscillator imposes temporal order on posterior gap gene expression in Drosophila
Source: PLoS Biol. 2018 Feb 16;16(2):e2003174. doi: 10.1371/journal.pbio.2003174 (PMC5832388; doi:10.1371/journal.pbio.2003174)

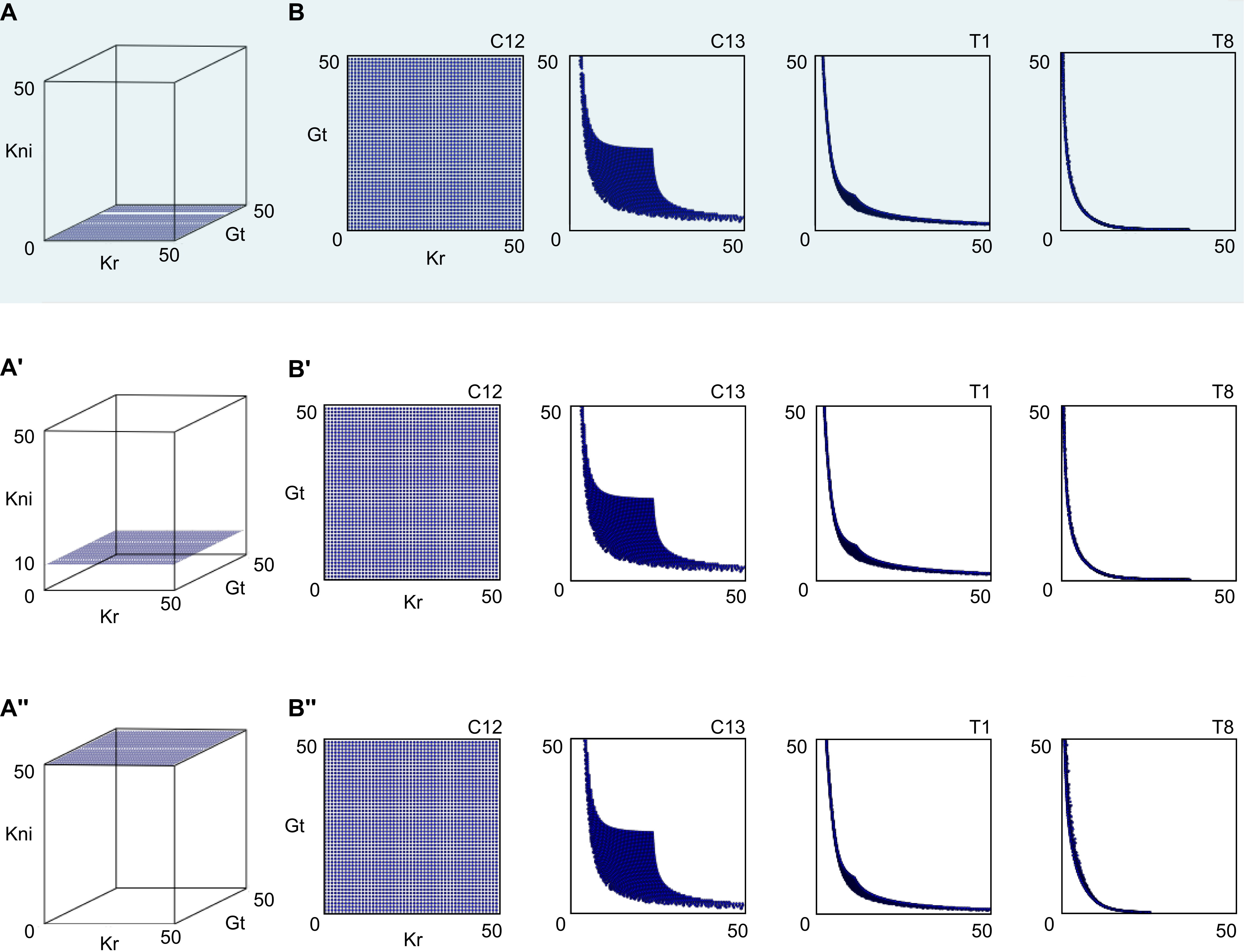

Supplement: S1 Fig — We simulate the nonautonomous diffusion-less circuit in the nucleus at 59% A–P position with Kni concentration fixed to zero (A, B), as in Fig 3A and 3B in the main manuscript, fixed to 10 (A′, B′) and fixed to 50 (A″, B″), and a set of initial conditions that are regularly distributed on the Kr-Gt plane. A–A″ Initial conditions shown in blue, embedded within the three-dimensional Kr-Kni-Gt space. B–B″ Two-dimensional projections of the Kr-Gt plane show converging system states (in blue) at the end of cleavage cycle 12 (C12), cleavage cycle 13 (C13), and cleavage cycle 14A (C14A, time classes T1 and T8). Concentrations in arbitrary units. See Materials and methods for time classes, and text of the main paper for further details. A–P, anteroposterior; au, arbitrary units; Gt, Giant; Kni, Knirps; Kr, Krüppel. (TIF) [file pbio.2003174.s001.tif]

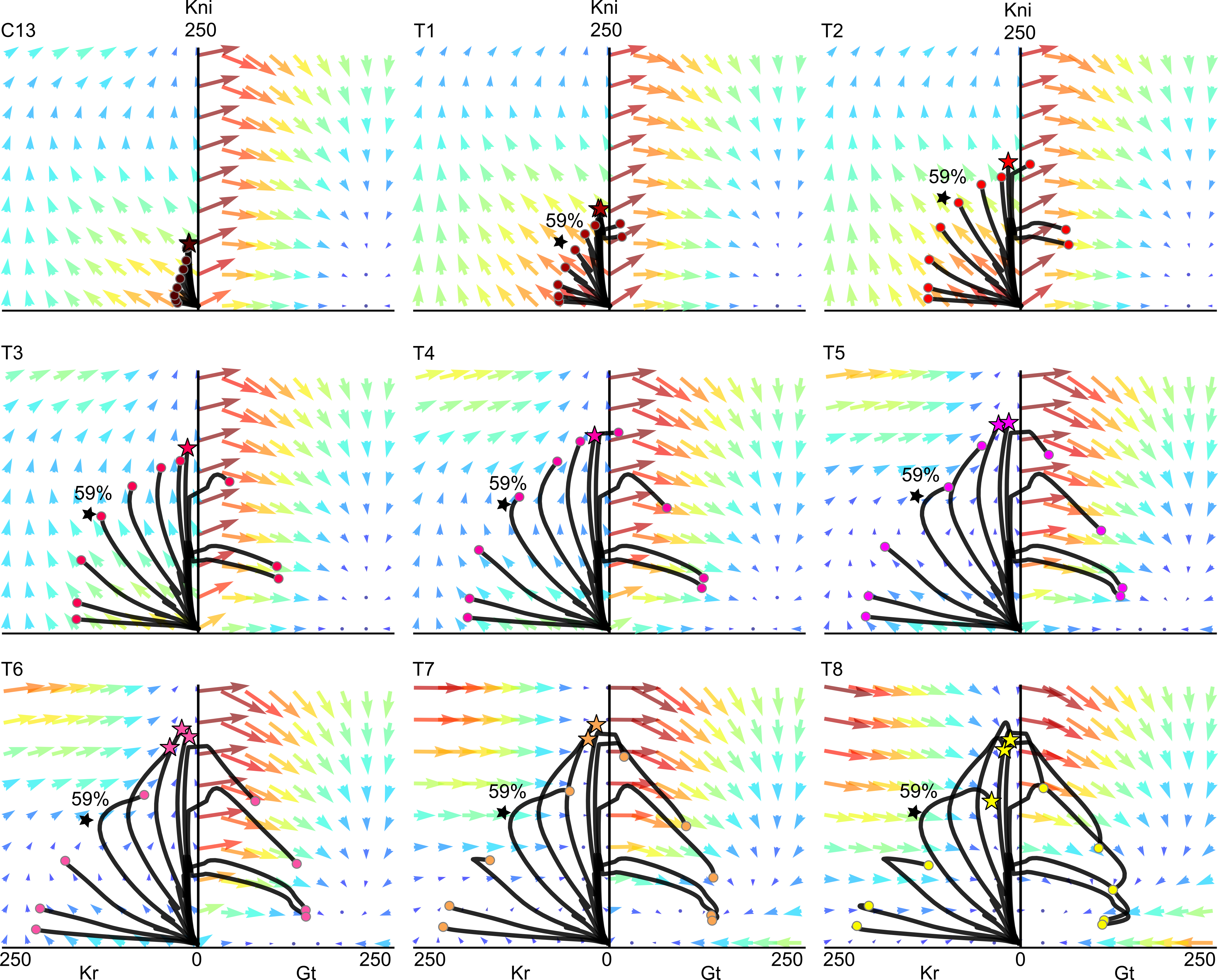

Supplement: S2 Fig — Fast-slow dynamics in posterior nuclei are caused by relaxation-like behavior. Unfolded, two-dimensional projections of the Kr-Kni and Kni-Gt planes are shown, as in Fig 3C of the main paper, at cleavage cycle 13 (C13) and cleavage cycle (C14A, time classes T1–T8). Colored arrows indicate magnitude and direction of flow: large red arrows represent strong flow; small blue arrows represent weak flow. Simulated trajectories of posterior nuclei are superimposed on the flow (shown as black lines). Small colored circles at the end of trajectories indicate current state at each time point (see key in Fig 2D of the main paper). Stars mark trajectories experiencing a positive Gt component of the flow. See main text for further details. Cad, Caudal; Gt, Giant; Kni, Knirps; Kr, Krüppel. (TIF) [file pbio.2003174.s002.tif]

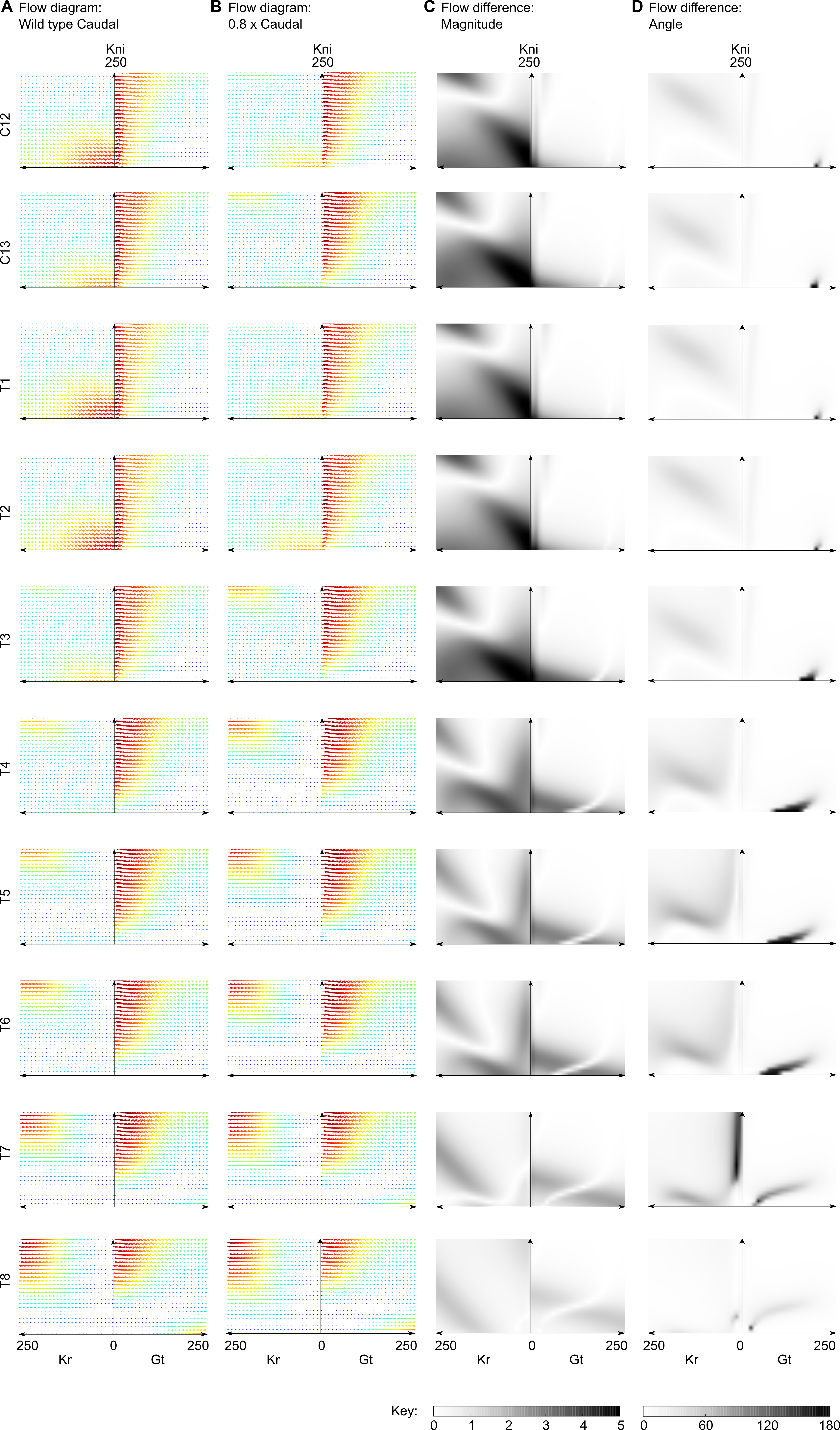

Supplement: S3 Fig — Unfolded, two-dimensional projections of the Kr-Kni and Kni-Gt planes are shown, as in Fig 4C–4E in the main paper, for all time classes (C12 indicating the onset of cleavage cycle C13 at t = 0). A, B. Plots with colored arrows indicate flow in a simulation with WT levels of Cad (A) or Cad levels scaled by a factor of 0.8 (B). C, D. Gray shading indicates differences of flow magnitude (C) and direction (D) (see keys). See main text for further details. Cad, Caudal; Gt, Giant; Kni, Knirps; Kr, Krüppel; WT, wild-type. (TIF) [file pbio.2003174.s003.tif]

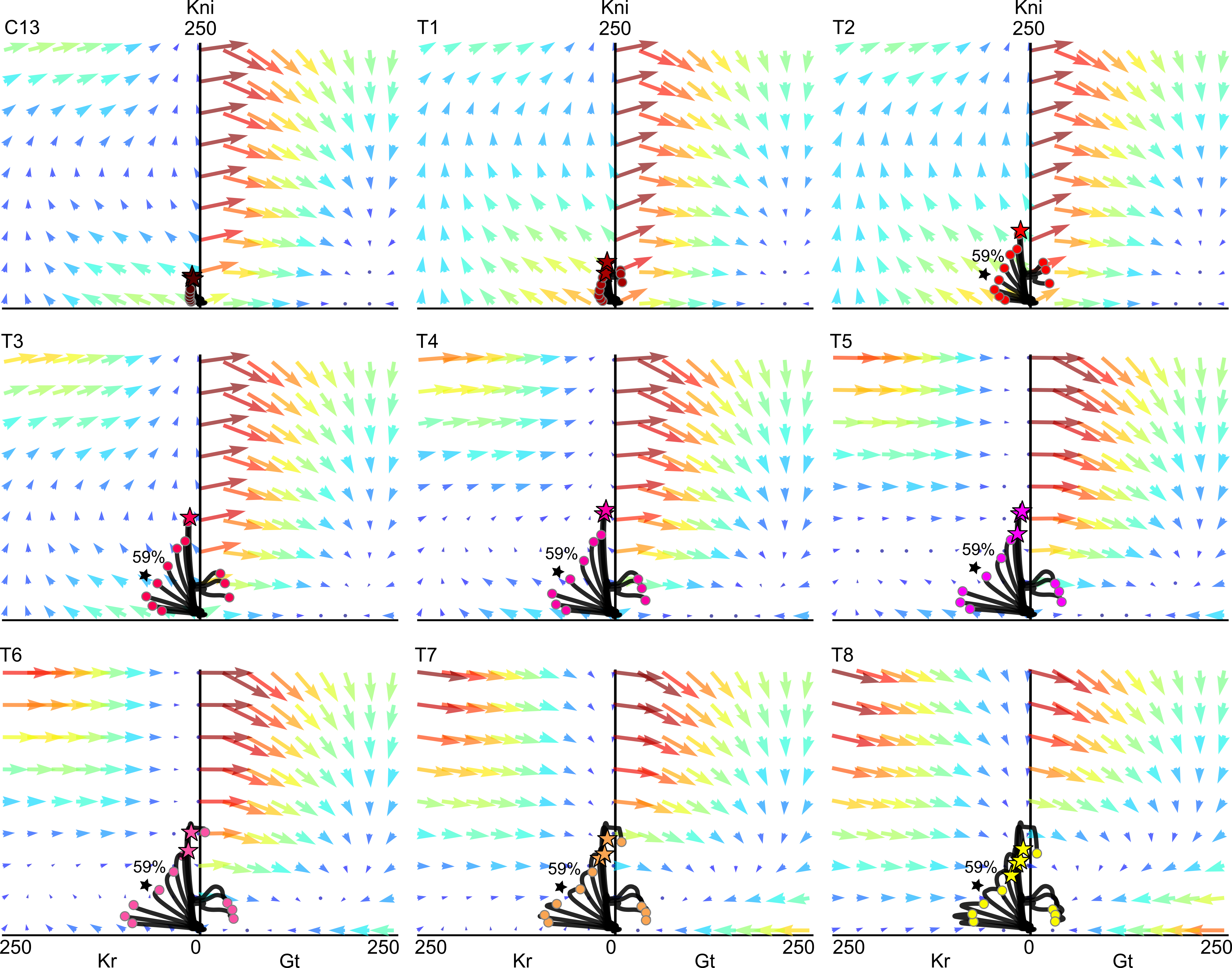

Supplement: S4 Fig — Fast-slow dynamics in posterior nuclei, caused by relaxation-like behavior, are robust to reduction of Cad concentration by a factor of 0.8. Unfolded, two-dimensional projections of the Kr-Kni and Kni-Gt planes are shown, as in Fig 3C (main paper), at cleavage cycle 13 (C13) and cleavage cycle 14A (C14A, time classes T1–T8). Colored arrows indicate magnitude and direction of flow: large red arrows represent strong flow; small blue arrows represent weak flow. Simulated trajectories of posterior nuclei are superimposed on the flow (shown as black lines). Small colored circles at the end of trajectories indicate current state at each time point (see key in Fig 2D of the main paper). Stars mark trajectories experiencing a positive Gt component of the flow. See main text for further details. Cad, Caudal; Gt, Giant; Kni, Knirps; Kr, Krüppel. (TIF) [file pbio.2003174.s004.tif]

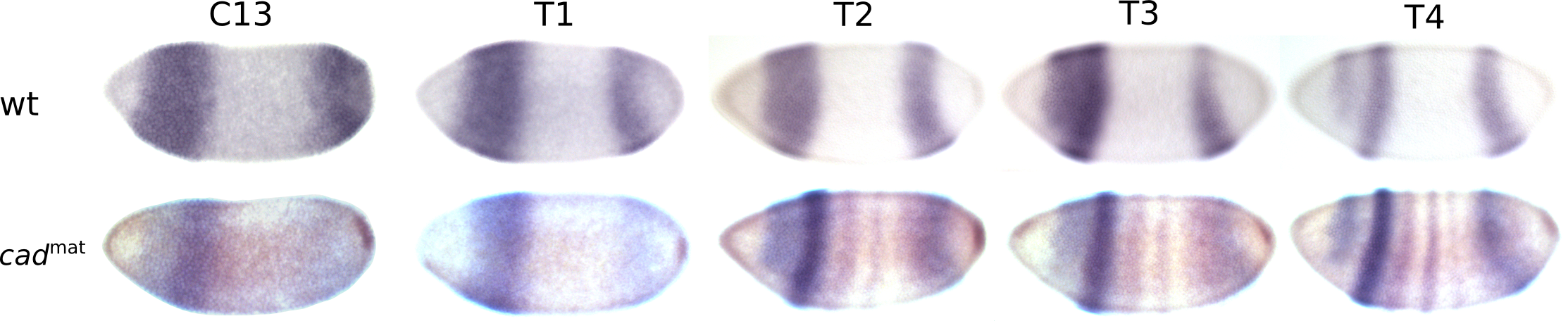

Supplement: S5 Fig — Laterally aligned embryos are shown (anterior is to the left, dorsal on top), stained by enzymatic (colorimetric) in situ hybridization for gt (blue), and also the pair-rule gene eve (red), in the case of cadmat embryos. Embryos are shown at cleavage cycle 13 (C13), and cleavage cycle 14A (C14A, time classes T1–T4), as indicated. Note that red background has been removed from double-stained cadmat embryos to emphasize the blue gt profiles in this figure. Parts of this data set were previously published in [39]. cadmat, maternal cad mutant; eve, even-skipped; gt, giant. (TIF) [file pbio.2003174.s005.tif]

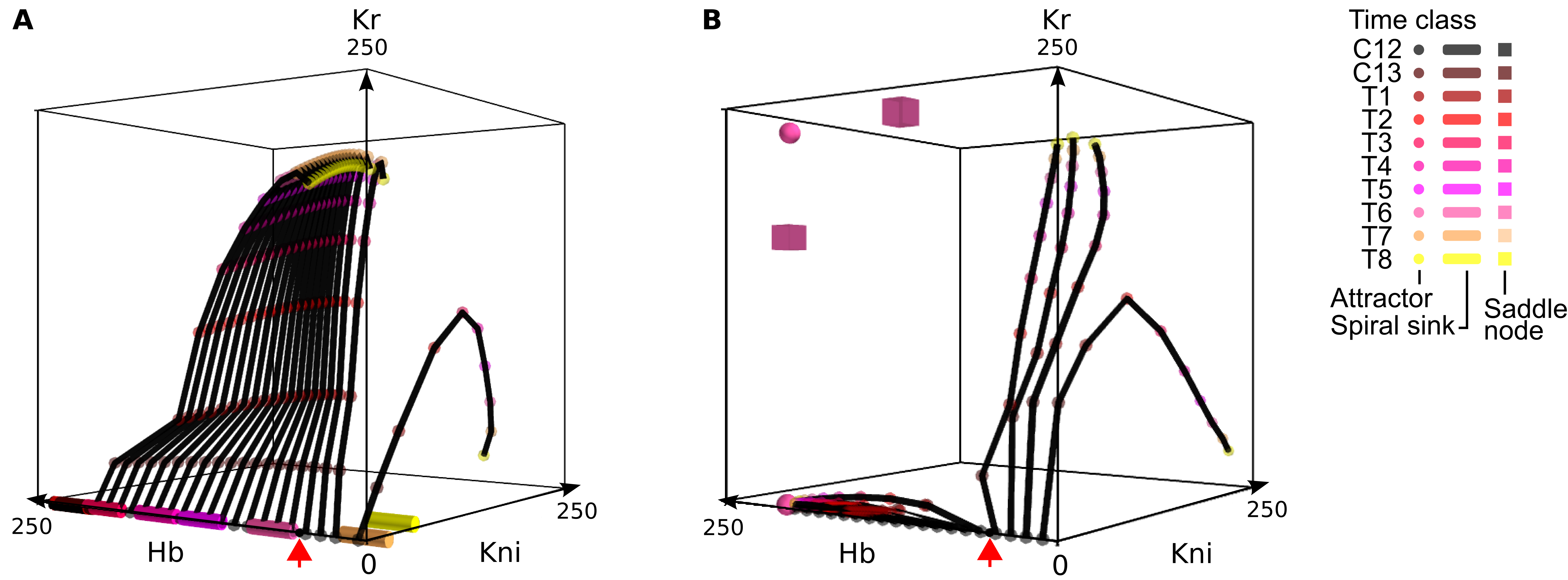

Supplement: S6 Fig — We use our model (A) and the static-Bcd model from [23] (B) to simulate 20 trajectories, with initial Hb concentrations ranging from 0 to 200 (arbitrary units). This mimics increasing levels of hb overexpression. Phase portraits are shown for the nucleus at 59% A–P position projected onto the three-dimensional subspace defined by Hb (x-axis), Kr (y-axis), and Kni (z-axis). Trajectories are shown as black lines. Spiral sinks are represented by cylinders, attractors by spheres, and saddle nodes by cubes. Small colored dots on trajectories indicate time points (see key for color coding). Red arrows indicate Hb = 44.04, marking the threshold above which trajectories converge directly towards their attractor instead of deviating through an unstable manifold in [23]. Trajectories that start above this threshold converge to a state with high Hb and Kr in (A) but to a state with high Hb only in (B). Phase space features shown in (B) correspond to those shown in Fig 4B of [23] to facilitate comparison. Saddles in (B) have one positive and three negative eigenvalues, indicating the presence of one-dimensional unstable manifolds. A–P, anteroposterior; Bcd, Bicoid; Hb, Hunchback; Kni, Knirps; Kr, Krüppel. (TIF) [file pbio.2003174.s006.tif]
