# Supplementary material for: A damped oscillator imposes temporal order on posterior gap gene expression in Drosophila
Source: PLoS Biol. 2018 Feb 16;16(2):e2003174. doi: 10.1371/journal.pbio.2003174 (PMC5832388; doi:10.1371/journal.pbio.2003174)
Supplement: S1 Table — Model equations are shown in the Materials and methods section. Values of promoter thresholds were fixed to −2.5 during optimization. (PDF) [file pbio.2003174.s010.pdf]

Promoter Strengths		R^{hb}	R^{Kr}	R^{gt}	R^{kni}
		10.00	16.93	15.97	13.01
Interconnectivity Matrix (W)		hb	kr	gt	kni
	hb	0.01	0.00	0.02	-0.26
	kr	0.00	0.01	-0.27	-0.01
	gt	-0.05	-0.39	0.00	0.02
	kni	-0.18	0.00	-0.06	0.01
External Input Strengths (E)		bcd	cad	hkb	tll
	hb	0.08	0.00	0.17	-0.85
	kr	0.07	0.02	-3.13	0.00
	gt	0.09	0.03	-0.09	0.00
	kni	0.00	0.03	-0.72	0.00
Promoter Thresholds*		h^{hb}	h^{Kr}	h^{gt}	h^{kni}
		-2.5	-2.5	-2.5	-2.5
Protein Half Lives		Hb	Kr	Gt	Kni
		15.47	9.67	9.66	20.00

S1 Table: Values of the parameters in the non-autonomous gap gene circuit model. Model equations are shown in the "Models and Methods" section of the main paper. *Values of promoter thresholds were fixed to -2.5 during optimisation.
