# Supplementary material for: A damped oscillator imposes temporal order on posterior gap gene expression in Drosophila
Source: PLoS Biol. 2018 Feb 16;16(2):e2003174. doi: 10.1371/journal.pbio.2003174 (PMC5832388; doi:10.1371/journal.pbio.2003174)
Supplement: S1 Text — (PDF) [file pbio.2003174.s011.pdf]

S1 Text
A damped oscillator imposes temporal order on posterior gap gene expression in *Drosophila*

Berta Verd[a,b,c,*], Erik Clark[d], Karl R. Wotton[a,b], Hilde Janssens[a,b], Eva Jiménez-Guri[a,b], Anton Crombach[a,b] and Johannes Jaeger[a,b,c,e,f,g,*]

[a]EMBL/CRG Systems Biology Research Unit, Centre for Genomic Regulation (CRG), The Barcelona Institute of Science and Technology, Dr. Aiguader 88, 08003 Barcelona, Spain
[b]Universitat Pompeu Fabra (UPF), Barcelona, Spain
[c]Konrad Lorenz Institute for Evolution & Cognition Research (KLI), Klosterneuburg, Austria
[d]Department of Zoology, University of Cambridge, Cambridge, UK
[e]Wissenschaftskolleg zu Berlin (Wiko), Germany
[f]Complexity Science Hub (CSH) Vienna, Austria
[g]Center for Systems Biology Dresden (CSBD), Germany
[*]Co-corresponding authors: bv291@cam.ac.uk, yoginho@gmail.com

January 23, 2018

Diffusion-less non-autonomous gap gene circuits

The gap gene circuit model used here and in [1] is based on the connectionist model formalism first presented by Mjolsness *et al.* [2]. A brief overview and model equations are given in the "Materials and Methods" section of the main paper. Here, we will highlight two important aspects of the model that differ from gene circuits used in previous work [3, 4, 5].

Gene circuit models: full versus partial non-autonomy

The gap gene circuit used in [3, 4] does not fully take into account the time-dependence of maternal morphogen gradients. In order to enable analysis with standard analytical methods [6], the model was simplified as follows: the Bicoid (Bcd) gradient was modelled by a static exponential profile, based on an

approximation of the Bcd concentration profile with an exponential function at cleavage cycle 13 (C13); Caudal (Cad) concentration was simulated using interpolated spatio-temporal profiles for time classes from C13 to C14A-T6. Phase space analysis was performed only at T6, using the Cad profile at this time point. In contrast, our model and analysis considers time-variable Bcd and Cad profiles for the entire duration of the blastoderm stage.

In mathematical terms, the model used in [3, 4] implements the contribution of maternal regulatory inputs to gap gene a in nucleus i by a term $e^{Bcd}g_i^{Bcd} + e^{Cad}g_i^{Cad}(t)$, where g_i^{Bcd} is the approximated exponential Bicoid profile at C13, and $g_i^{Cad}(t)$ is derived from interpolated data up until C14A-T6, and held constant at the level of T6 afterwards. On the one hand, this simplification is justified in that it allows for autonomous model analysis of the pattern formed at the late blastoderm stage. On the other hand, it means that this version of the model is not suited to study the effects of time-variable maternal inputs—especially gradient decay during the late blastoderm.

For this reason, we have made the model more realistic and accurate by including the full temporal dynamics of maternal gradients [1]. In our model, the contribution of maternal regulatory inputs to gap gene a in nucleus i is given by the term $\sum_{m \in M} E^{ma} g_i^m(t)$ (see Equation 3 in the main paper) where $M = \{\text{Bcd}, \text{Cad}\}$. Here, $g_i^m(t)$ are both derived from interpolated spatio-temporal expression data for all time classes. The explicit time dependence of $g_i^m(t)$ makes our model fully non-autonomous, which requires novel methods for phase space analysis (see Model Analysis section in the main paper) [7].

Diffusion-less model formulation

Our analysis requires a diffusion-less model to reduce the dimensionality of phase space from 160 to four dimensions, to render visualisation tractable. The model presented in this study (and in [1]) is a diffusion-less version of the non-autonomous gap gene circuits published in [5]. Even though diffusion of gap proteins occurs in *Drosophila* embryos, it is not required for gap domain placement and shifts; instead it is only involved in smoothing spatial boundaries of gap domains in gene circuit models [8, 4]. This allows us to eliminate diffusion from our model without affecting its validity for the analysis of regulatory mechanisms underlying pattern formation.

Model Fitting

Network structure

The gap gene system is one of the most thoroughly studied gene regulatory networks in developmental biology today. Its regulatory structure has been determined by decades of extensive genetic and molecular studies (reviewed in [9]), and data-driven modelling approaches using gene circuits [8, 10, 3, 4, 11, 12, 13, 5, 14]. These studies have yielded an experimentally verified consensus network structure of activating and repressing regulatory interactions that is very well established [9]. Considering this state of the art, it is not our aim here to gain new insights into interactions between gap genes through reverse-engineering. Instead, we want to analyse gap gene circuits that adhere to the established network structure in order to facilitate comparison with earlier experimental and modelling work. For this reason, we constrain the qualitative nature of regulatory interactions (whether they are activating or repressing) according to the consensus network structure implemented in previous models [4, 5]. Only the strength of each interaction—encoded by the numerical values of regulatory parameters—was allowed to vary during the model fitting procedure. Threshold parameters h_a were fixed to negative values; we have shown previously that this increases optimization efficiency without affecting the quality of the resulting fit [5, 14].

Model fitting and selection

Model fitting was performed as described in detail in [1]. The full fitted model covers a spatial domain between 35 and 92% A–P position, and includes Hb, Kr, Kni, and Gt as state variables as well as Bcd, Cad, and the terminal gap gene products Tll and Hkb as external inputs. The model was fit without gap protein diffusion. We numerically solve Equation 1 from the "Model and Methods" section of the main paper with a Runge-Kutta Cash-Karp adaptive step-size solver [5]. We compare model output to a previously published quantitative data set of spatio-temporal gap protein expression [15, 16, 5]. The difference between model output and data is minimized using global optimization by parallel Lam Simulated Annealing (pLSA) [17] with a weighted least squares cost function [5]. Optimization runs were performed on the Mare Nostrum supercomputer at the Barcelona Supercomputing Centre (http://www.bsc.es). Each optimization run lasted around 35 minutes on 64 cores. We obtained 200 fitted gene circuits. From this set of solutions, we selected a small number of best-fitting circuits for further analysis based on the following criteria: (a) numerically unstable circuits were discarded; (b) fits with a root-mean-square score (RMS) above 20.0 were discarded; (c) fits with gross patterning defects were eliminated by visual inspection as described in detail in [14, 1]. This resulted in a set of three satisfactorily accurate fitting solutions. We used one of these three circuits for our analysis (parameters that are relevant in the spatial domain of our current analysis are shown in S1 Table. The other two also show oscillatory features in

the posterior of the embryo, but their phase spaces are less structurally stable than the circuit analysed in depth.

The residual error of the non-autonomous diffusion-less circuit used here is at a weighted root mean square (RMS) score of 14.53. A detailed comparison to non-autonomous circuits with diffusion, as well as static-Bcd models is provided in [1]. To briefly summarize: the score of our circuit lies very close to the upper end of the range of residual errors for fully non-autonomous circuits with diffusion (10.43–13.32). This indicates that switching off diffusion does not substantially affect fitting quality. Even though minor deviations of domain boundary shape can be observed at 43/44% and 65/66% A–P position, the timing and relative spatial positioning of domains is faithfully reproduced (see Fig. 1A of the main paper). Model fits using weighted RMS are more accurate than those using un-weighted data [5], even though un-weighted RMS scores are generally lower (8.71–10.11 for circuits with diffusion). The diffusion-less static-Bcd circuit used in [4] has a somewhat higher RMS of 10.76. This suggests that the effects of switching off diffusion are similar, and minor, for both static-Bcd and non-autonomous models.

After fitting, we tested the effect of adding diffusion back into our circuit in two different ways. First, we combined fitted values for diffusion rates d^a from a non-autonomous circuit fitted with diffusion. In this circuit the sign of the regulatory parameters were kept the same as in our diffusion-less fit (which is the same as in [4]). These parameters fall into a range of 0.19 to 0.28. This increases the RMS score to 19.96, mainly due to significantly lower levels of Gt both in its anterior and posterior domains, while the relative timing and arrangement of gap domains is maintained. We then tested the effect of diffusion in a second way, by increasing the values of d^a by small increments in our diffusion-less circuit. This shows that patterning defects only arise above diffusion rates of 0.1 (about half the value of the fitted rates). Again, higher diffusion rates cause lower levels of Gt in the anterior and the posterior, but cause no major patterning disruption in the timing and arrangement of gap domains.

Glossary of dynamical systems concepts

Attractor:

These are states (or a set of states) of the system that *trajectories* in their vicinity converge to. Attractors can be single points like *point attractors* and *spiral sinks*, but can also refer to attractive sets like *limit cycles*.

Autonomous dynamical system:

A *dynamical system* whose right-hand side (especially its *parameters*) remain constant over time.

Basin of attraction:

The region of *phase space* surrounding an *attractor*, and defined by the totality of *trajectories* that converge towards it.

Bifurcation:

An event during which the structure of the system's flow changes qualitatively in response to parameter changes. Steady states can change their nature (*e. g.* stable to unstable), and/or be created or annihilated. A bifurcation changes the topology of *phase space* by changing the number and/or type of *steady states* it contains. Often, bifurcations will also change the basic geometric arrangement of attractors and their basins with respect to each other.

Boundary conditions:

These represent external inputs to the system. In our case, the dynamically changing maternal gradients of Bcd and Cad are the boundary conditions of the system. Boundary conditions can be considered *parameters* of the system in this case, since they are not themselves affected by the *system state*.

Damped oscillator:

A damped oscillator is mathematically defined as a *dynamical system* driven by a *spiral sink*. This definition applies, even if the system does not exhibit periodically repeating patterns of states. Damped oscillators belong to the broader class of *dynamical systems* known as *oscillators*, which, in brief, impose stereotypical order onto all of their trajectories. In our case, system *trajectories* spiralling towards a mono-stable *spiral sink* in the posterior region of the embryo lead to a stereotypical temporal progression of gap gene expression. In this paper we show that, if properly arranged in space, this produces kinematic gap domain shifts toward the anterior. Therefore, gap domain shifts are driven by a damped oscillator in our *non-autonomous* gap gene circuit.

Dynamical regime:

We define a dynamical regime by the topology of the *phase portrait* (the number, type and geometrical arrangement of its attractors and their basins), and the range of parameters over which a particular topology persists. They can be used to classify the possible behaviours of *dynamical systems*. In the context of our gap gene circuit analysis, we use the term to distinguish multi-stable dynamics (governed by the number and position of *attractors* in *phase space*) from oscillatory dynamics (in our case, governed by spiralling transient system *trajectories* far from steady state). Different *parameters* and *phase space* geometries can drive the same type of dynamics, especially when these are the result of transient trajectories, and therefore operate in the same dynamical regime.

Dynamical system:

A system of mathematical equations (in our case: ordinary differential equations), which describes how a given set of *state variables* change over time according to given rules (which define the regulatory structure of the system). Dynamical systems can be classified into *autonomous dynamical systems* (where the right-hand side of the equation is not explicitly time dependent, and *parameters* remain constant over time), and *non-autonomous dynamical systems* (where the right-hand side of the equation is explicitly time-dependent, and *parameter* values are allowed to change over time).

Eigenvalue analysis:

We perform eigenvalue analysis to determine the stability and type of a given steady state. This will tell us whether the steady state is stable or unstable, and also allow us to differentiate between certain types of steady states that have the same stability *e. g.* between a *point attractor* and a *spiral sink*. To find the eigenvalues associated with a given steady state we perform linear stability analysis around this steady state. In what follows we will illustrate the procedure in two dimensions. Let's consider the following two dimensional system of differential equations:

$$\dot{x} = f(x, y) \tag{1}$$

$$\dot{y} = g(x, y), \tag{2}$$

and let's suppose that $(\bar{x}, \bar{y})$ is a steady state such that $f(\bar{x}, \bar{y}) = 0$ and $g(\bar{x}, \bar{y}) = 0$. To know whether the steady state is stable or unstable we consider a small perturbation around the steady state such that

$$x = \bar{x} + u \tag{3}$$

$$y = \bar{y} + v, \tag{4}$$

where u and v are small. To determine whether u and v will grow and x and y will diverge from the steady state $(\bar{x}, \bar{y})$, or on the contrary u and v will shrink

and x and y will converge to the steady state $(\bar{x}, \bar{y})$ we derive the differential equations for u and v. This will ultimately tell us whether the steady state is stable or unstable.

$$\begin{aligned}
\dot{u} &= \dot{x} && (\bar{x} \text{ is constant}) \\
&= f(x, y) && (\text{by definition}) \\
&= f(\bar{x} + u, \bar{y} + v) && (\text{by substitution}) \\
&= f(\bar{x}, \bar{y}) + \frac{\partial f}{\partial x}(\bar{x}, \bar{y})u + \frac{\partial f}{\partial y}(\bar{x}, \bar{y})v + ... && (\text{Taylor series}) \\
&= \frac{\partial f}{\partial x}(\bar{x}, \bar{y})u + \frac{\partial f}{\partial y}(\bar{x}, \bar{y})v + ... && (\text{since } f(\bar{x}, \bar{y}) = 0 \text{ })
\end{aligned}$$

The same is true for v:

$$\dot{v} = \frac{\partial g}{\partial x}(\bar{x}, \bar{y})u + \frac{\partial g}{\partial y}(\bar{x}, \bar{y})v + ... \tag{5}$$

Higher order terms involving u^2, v^2, uv ... can be neglected since they are assumed to be very small because u and v are already small. We now have a linear system describing the evolution of the perturbations u and v over time:

$$\begin{pmatrix} \dot{u} \\ \dot{v} \end{pmatrix} = \begin{pmatrix} \frac{\partial f}{\partial x}(\bar{x}, \bar{y}) & \frac{\partial f}{\partial y}(\bar{x}, \bar{y}) \\ \frac{\partial g}{\partial x}(\bar{x}, \bar{y}) & \frac{\partial g}{\partial y}(\bar{x}, \bar{y}) \end{pmatrix} \begin{pmatrix} u \\ v \end{pmatrix} \tag{6}$$

The 2x2 matrix in equation 6 is known as the Jacobian. Its associated eigenvalues are given by solving the characteristic equation for λ:

$$det(J - \lambda I) = 0 \tag{7}$$

where I is the 2x2 identity matrix. The same procedure is used to calculate eigenvalues in higher dimensional systems, like for example, the four dimensional gap gene system.

Knowing the real parts of the eigenvalues gives us information about the direction of the *flow* around a *steady state*, and therefore its stability. If all the eigenvalues of the Jacobian have negative real parts, the steady state is stable. On the other hand, if at least one of the eigenvalues has a positive real part, the steady state is unstable. The imaginary parts of the eigenvalues tell us whether convergence (or divergence) is linear or spiralling. For example, *attractors* have real negative eigenvalues (no imaginary parts) while *spiral sinks* have eigenvalues with negative real parts, of which at least two are conjugate pairs with non-zero imaginary parts; *saddle points* have at least one eigenvalue with positive real part.

Flow:

The rate of change of the system at all possible *states*. Also the sum total of all possible *trajectories* in a system.

Initial condition:

The *state of the system* at $t = 0$. In our case, initial conditions consist of non-zero concentration of maternal Bcd, Cad, and Hb, as well as zero concentrations of trunk gap gene products Kr, Kni, and Gt.

Instantaneous phase portrait:

The *phase portrait* associated with a *non-autonomous dynamical system* changes over time due to changes in the values of systems *parameters*. We visualise the dynamic geometry of *phase space* in these systems through snapshots of the *phase portrait* at different points in time. This is achieved through instantaneous phase portraits, which are created by "freezing" the values of time-dependent parameters at a given time, and then plotting the *phase portrait* of the resulting *autonomous dynamical system*.

Limit cycle:

A limit cycle is a closed trajectory in phase space which attracts or repels trajectories in its vicinity. A stable limit cycle draws system *trajectories* towards it, and is in this sense an *attractor* set. Limit cycles underlie sustained oscillations, such as those observed in gene expression involved in short-germband segment determination and vertebrate somitogenesis.

Non-autonomous dynamical system:

A *dynamical system* where the right hand side is explicitly time dependent, for example if at least one of the *parameters* is time-dependent.

Oscillator:

Refers to a general class of *dynamical systems* which have the property of imposing a stereotypical order onto the *trajectories* of the system. Examples of oscillators include periodic oscillators (also known as *limit cycle* oscillators), conservative-free oscillators (initial condition-dependent periodic oscillators), coupled oscillators, chaotic oscillators, *relaxation oscillators* and of course, *damped oscillators*. Oscillators are not defined by the existence of a *limit cycle*. This is nicely exemplified by conservative-free oscillators where for every *initial condition* there is a closed, periodic trajectory (orbit), but no *limit cycle*. However, the opposite is true: a *limit cycle* implies an oscillator. Mono-stable *dynamical systems* where the *steady state* has at least one pair of complex *eigenvalues* are often oscillators (*e. g.* unstable *spiral sinks* co-exist with stable *limit cycles*).

Parameters:

In contrast to the *state variables*, system parameters describe those factors that do not change in response to the system's state. They can remain constant

over time (*autonomous dynamical systems*), or change in response to external influences (*non-autonomous dynamical systems*). In our case, parameters define production and decay rates, and genetic interactions between gap genes, as well as the regulatory input from maternal factors.

Phase portrait:

The graphical representation of *phase space* for a particular *dynamical system*. Represents features of phase space such as *trajectories*, the *flow*, *limit cycles*, *steady states* (*e. g. attractors*, *spiral sinks*, or *saddle points*), and *basins of attraction* bounded by *separatrices*. The phase portrait remains constant over time for *autonomous dynamical systems*, but is time-variable for *non-autonomous dynamical systems*.

Phase (or state) space:

An abstract space whose axes are given by the values of *state variables* of the system (in our case, the concentrations of the trunk gap genes *hb*, *Kr*, *kni*, and *gt*). It represents all possible *states* of the system.

Point attractor:

A state of the system towards which trajectories converge in straight lines. The *eigenvalues* associated with attractor points are real and negative.

Relaxation oscillator:

A *dynamical system* that drives sustained oscillatory dynamics that show an alteration of a slow, gradual build-up in some state variables with rapid, almost explosive, release and change in state. These are called relaxation oscillations. They are caused by an irregularly shaped *limit cycle*. Irregularly shaped spiralling trajectories show similar dynamic behaviour, which we call "relaxation-like."

Saddle point:

An unstable *steady state*. Always lies on a *separatrix*. Only *trajectories* exactly on the *separatrix* will converge to this steady state; local perturbations that take *trajectories* off the *separatrix* will cause divergence towards a stable steady state, typically along a *trajectory* called an *unstable manifold*.

Separatrix:

The boundary of a *basin of attraction* separating trajectories that converge to different stable *steady states*. *Saddle points* always lie on separatrices.

Spiral sink (or focus):

Similar to *point attractors*, spiral sinks are stable *steady states* that draw system *trajectories* towards them. Unlike *point attractors*, *trajectories* do not converge towards a sink in straight lines, but rather spiral inwards. Eigenvalues associated with spiral sinks have negative real parts and include at least one complex conjugate pair. Spiral sinks are the hallmark of *damped oscillators*, as the spiralling geometry of the *flow* imposes a stereotypical temporal succession of states onto the trajectories of the system. (See also *oscillator* above.)

State variables:

In contrast to system *parameters*, state variables represent those components of the system whose values change over time due to the influence of other state variables and external inputs (*boundary conditions*) to the system. In our case, the state variables represent the concentration of trunk gap gene products Hb, Kr, Kni, and Gt.

Steady state:

This is a state of the system at which the *flow* is zero. Once the system has reached a steady state, no further change in state occurs (unless the system is perturbed). Steady states can be classified into stable (*e. g. attractors*, or *spiral sinks*) and unstable steady states (*e. g. saddle points*). At stable steady states, the system re-converges upon local perturbation; at unstable steady states, the system diverges to a stable steady state upon perturbation.

System state:

Defined by the value of the *state variables* at a given time: for example, concentration of trunk gap gene products Hb, Kr, Kni, and Gt at developmental stage C14A-T3.

Trajectory:

A trajectory of the system describes the temporal evolution of the *state variables* given specific *initial* and *boundary conditions.* In our case, a trajectory describes the change in concentration of trunk gap gene products in a given nucleus over time. Here, the initial and boundary conditions are given by the concentrations of maternal morphogens Bcd, Cad, and Hb.

Unstable manifold:

A *trajectory* in *phase space* that links a *saddle point* to an *attractor.*

References

[1] Verd B, Crombach A, Jaeger J. Dynamic Maternal Gradients Control Timing and Shift-Rates for *Drosophila* Gap Gene Expression. PLOS Computational Biology. 2017;13:e1005285.

[2] Mjolsness E, Sharp DH, Reinitz J. A connectionist model of development. The Journal of Theoretical Biology. 1991;152:429–53.

[3] Manu, Surkova A, Spirov A, Gursky V, Janssens H, Kim A-R, Radulescu O, Vanario-Alonso CE, Sharp DH, Samsonova M, Reinitz J. Canalization of Gene Expression in the *Drosophila* Blastoderm by Gap Gene Cross Regulation. PLoS Biology. 2009;7:e1000049.

[4] Manu, Spirov AV, Gursky VV, Janssens H, Kim AR, Radulescu O, Vanario-Alonso CE, Sharp DH, Samsonova M, Reinitz J. Canalization of gene expression and domain shifts in the *Drosophila* blastoderm by dynamical attractors. PLoS Computational Biology. 2009;5:e1000303.

[5] Ashyraliyev M, Siggens K, Janssens H, Blom J, Akam M, Jaeger J. Gene circuit analysis of the terminal gap gene *huckebein*. PLoS Computational Biology. 2009;5:e1000696.

[6] Strogatz SH. Nonlinear Dynamics and Chaos: with Applications to Physics, Biology, Chemistry, and Engineering. Boulder. CO: Westview Press; 2014.

[7] Verd B, Crombach A, Jaeger J. Classification of transient behaviours in a time-dependent toggle switch model. BMC Systems Biology. 2014;8:43.

[8] Jaeger J, Surkova S, Blagov M, Janssens H, Kosman D, Kozlov KN, Manu, Myasnikova E, Vanario-Alonso CE, Samsonova M, Sharp DH, Reinitz J. Dynamic control of positional information in the early *Drosophila* embryo. Nature. 2004;430:368–71.

[9] Jaeger J. The gap gene network. Cellular and Molecular Life Sciences. 2011;68:243–274.

[10] Jaeger J, Blagov M, Kosman D, Kozlov KN, Myasnikova E, Surkova S, Vanario-Alonso CE, Samsonova M, Sharp DH, Reinitz J. Dynamical analysis of regulatory interactions in the gap gene system of *Drosophila melanogaster*. Genetics. 2004;167:1721–37.

[11] Perkins T, Jaeger J, Reinitz J, Glass L. Reverse engineering the gap gene network of *Drosophila melanogaster*. PLoS Computational Biology. 2006;2:e51.

[12] Jaeger J, Sharp DH, Reinitz J. Known maternal gradients are not sufficient for the establishment of gap domains in *Drosophila melanogaster*. Mechanisms of Development. 2007;124:108–28.

[13] Gursky VV, Kozlov K, Samsonov AM, Reinitz J. Model with asymptotically stable dynamics for *Drosophila* gap gene network. Biophysics. 2008;53:164–76.

[14] Crombach A, Wotton KR, Cicin-Sain D, Ashyraliyev M, Jaeger J. Efficient reverse-engineering of a developmental gene regulatory network. PLoS Computational Biology. 2012;8:e1002589.

[15] Surkova S, Kosman D, Kozlov K, Myasnikova E, Samsonova AA, Spirov A, Vanario-Alonso CE, Samsonova M, Reinitz J. Characterization of the *Drosophila* segment determination morphome. Developmental Biology. 2008;313:844–62.

[16] Pisarev A, Poustelnikova E, Samsonova M, Reinitz J. FlyEx, the quantitative atlas on segmentation gene expression at cellular resolution. Nucleic Acids Research. 2009;37(Suppl 1):D560–6.

[17] Chu KW, Deng Y, Reinitz J. Parallel simulated annealing by mixing of states. Journal of Computational Physics. 1999;148:646–62.
